# Supplementary material for: Changes in Yield-Related Traits, Phytochemical Composition, and Antioxidant Activity of Pepper (Capsicum annuum) Depending on Its Variety, Fruit Position, and Ripening Stage
Source: Foods. 2023 Oct 29;12(21):3948. doi: 10.3390/foods12213948 (PMC10648119; doi:10.3390/foods12213948)
Supplement: Supplementary file 1 [file foods-12-03948-s001.zip › foods-2600322-Supplementary Materials.pdf]

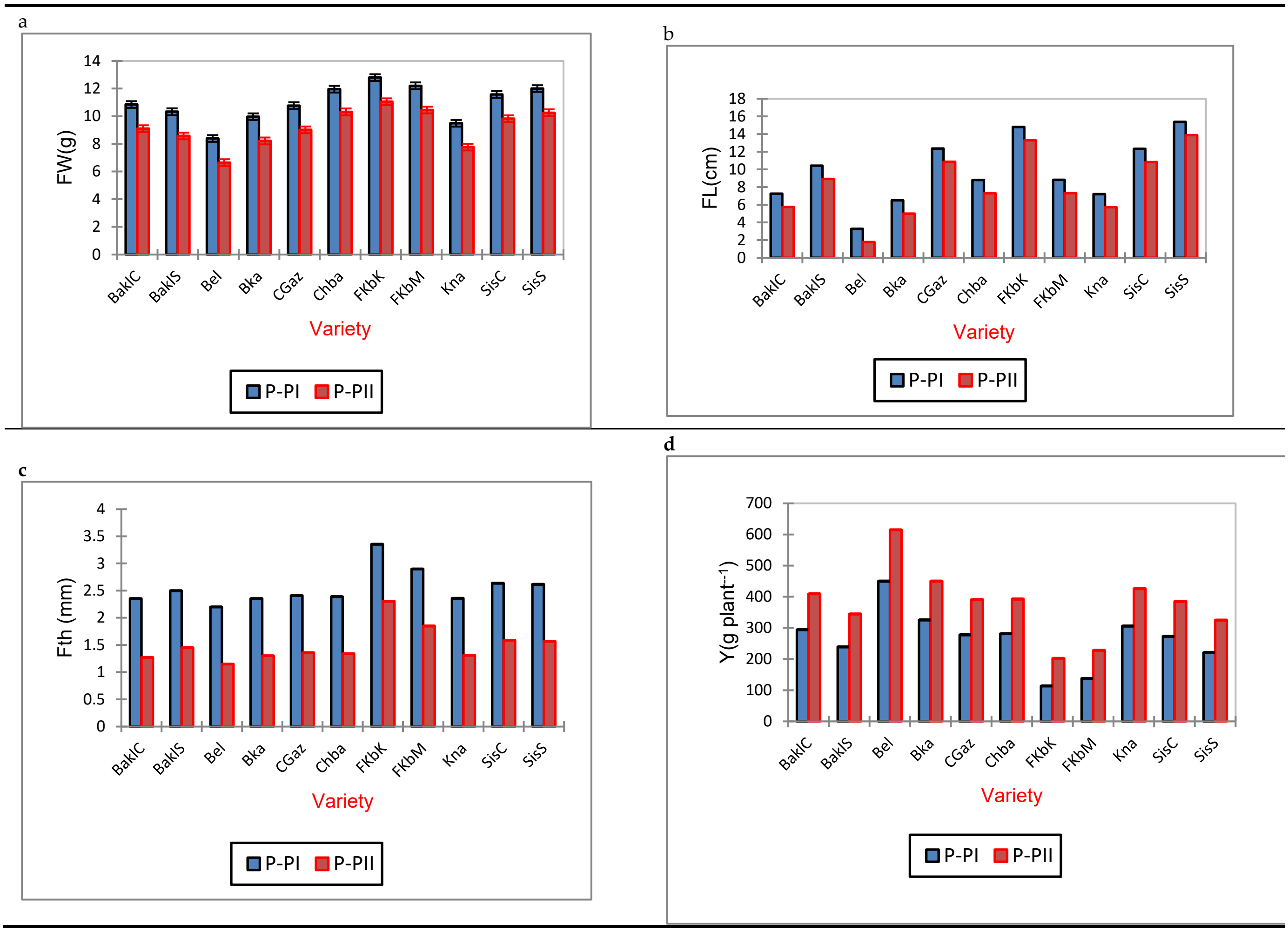

Figure S1 'Variety x fruit position' interaction on morphometric traits for the studied traits in eleven Tunisian chili pepper. BaklC: Baklouti Chébika ; Bel: Beldi ; Chba: Chaabani ; SisC: SissebChébika ; Bka: Bkalti ; Kna knaiss ; BaklS: BakloutiSbikha ; SisS: SissebSbikha ; FkbM: Fort Menzeltemim ; FkbK: Fort de korba and CGaz: Corne de Gazelle ; FW: Fruit weight; FL: fruit length; Fth: fruit thickness; Y: yield; PI: lower fruit position plant; PII: upper fruit position plant
